# Compressive Behaviour of Lattice Structures Manufactured by Polyjet Technologies

**DOI:** 10.3390/polym12122767

**Published:** 2020-11-24

**Authors:** Camil Lancea, Ian Campbell, Lucia-Antoneta Chicos, Sebastian-Marian Zaharia

**Affiliations:** 1 Department of Manufacturing Engineering, Faculty of Technological Engineering and Industrial Management, Transilvania University of Brasov, 500036 Brasov, Romania; l.chicos@unitbv.ro (L.-A.C.); zaharia_sebastian@unitbv.ro (S.-M.Z.); 2 Design School, Epinal Way, Loughborough University, Loughborough LE11 3TU, UK; R.I.Campbell@lboro.ac.uk

**Keywords:** additive manufacturing, material jetting, polymer, lattice structures, compressive tests, FEA

## Abstract

Additive manufacturing (AM) techniques can help to reduce the time and cost for manufacturing complex shaped parts. The main goal of this research was to determine the best strength structure of six different types of lattice cells, manufactured using the Poly Jet AM technology. In order to perform the tests, six samples with the same structure were created for each lattice type. For testing the samples in compression, an electromechanical test machine was used. finite element analysis (FEA) analysis was used in order to determine the area where the greatest stresses occured and to estimate the maximal compressive strength. The strongest structure was determined by obtaining the maximal compressive strength. This was calculated in two ways: as a ratio between the maximal supported force and the mass of the sample (N/g) and as a ratio between the maximal supported force and the critical section of the sample (MPa).

## 1. Introduction

Nowadays it is clear that world markets, and especially the European market, are in a continuous state of development and transformation. At the same time, due to globalization, the competition amongst product manufacturers, within the European Union, has become very intense. It is also well-known that protection of the environment is one of the top global priorities, as it has a major impact on the entire population of the planet. Among the measures to be taken in this regard are saving energy resources, reducing pollution, reducing the consumption of materials, recycling and reusing materials, and using renewable energy sources.

However, it is still important to maintain or even improve the quality of new products by increasing their resistance to various mechanical, dynamic, thermal, and chemical stresses, while, at the same time, minimising their cost. One of the solutions that meets many of the above requirements is the replacement of solid, dense materials with new cellular materials that offer high strength-to-mass and stiffness-to-mass ratios.

Traditionally, the materials in many products are, for the most part, solid and completely dense, and fully comply with the requirements to make the product reliable. In this regard, a number of features such as surface hardness, wear resistance, fatigue strength, corrosion resistance, thermal resistance etc. are very important. Fernandes [1] compared the mechanical performance of agglomerated cork against synthetic materials and it was discovered that the materials we find in nature, due to their cellular structure, offer a better capability for attaining this multi-functionality. Examples include the high strength-to-mass ratios seen in bone structures, honeycombs, insect wings, and tree structures.

In view of the above, a possible solution for obtaining products with a high resistance to various stresses, while allowing savings in material, is the use of lattice structures. Gibson [2] and Zangana [3] present the mechanical behaviour of different structures of cellular materials, including honeycombs and foams, made from polymers or a composite sandwich. Starting from the analysis of these structures, a new cellular structure was designed, by using the advantages of geometrical freedom that additive manufacturing (AM) gives.

The mechanical properties of cellular materials, such as yield stress or Young’s and Bulk moduli, are highly affected by the material architecture and volume fraction, as mentioned in the literature. Park [4] presents a two-step homogenization method, implemented for evaluating the mechanical properties of lattice structured material, fabricated by an additive manufacturing process. Mahshid [5] examines the effect of cellular lattice structures on the strength of the additively manufactured parts through compression tests applied to the specimens. The analytical approach includes finite element, geometrical and mathematical models for prediction of the collapse strength. Moronia [6] analysed the influence of different lattice structures on dynamic mechanical properties and Kadkhodapour [7] analysed the compression resistance of VeroBlue FullCure 840 photopolymer resin scaffold structures. Siddika [8] present a state-of-the-art review about the mechanical resistance of reinforced concrete structures.

AM is an advanced manufacturing technique which works by slicing a CAD model with parallel planes and using the profile data obtained to facilitate subsequent fabrication of a physical part, layer by layer. According to ISO/ASTM 52921, AM for polymer parts can be divided into four process categories, according to the techniques used to create the successive layers, as follows:

- Vat Photo Polymerization—uses a liquid photopolymer resin, located in the printer tank (composed of a liquid photopolymer to which a special hardener is added) which solidifies (polymerizes) in successive layers, under the influence of a laser with ultraviolet light, for obtaining the solid three-dimensional model. e.g., stereolithography (SLA), digital light processing (DLP).
- Material Jetting—works similarly to 2D printers. A print-head deposits droplets of a photosensitive material (photopolymer), which solidifies under ultraviolet (UV) light, for building the object layer by layer. e.g., PolyJet technology.
- Binder Jetting—uses a bed of powder, on which the nozzles spray micro fine drops of a liquid, which glues the powder particles to build a section of the piece. e.g., 3D Printing.

Initially, AM was used to make prototypes for visualization and assembly checking. Later, it was also used to make parts that would undergo aerodynamic and mechanical tests. Currently, AM technology has considerably broadened its utility scope, making it possible to be used for manufacturing tools and devices, for manufacturing functional components of products, or even for manufacturing products in their entirety. In this regard, Singha [9] presents a review of some of the most widely used AM techniques. Chu [10] explored the opportunities and challenges in design for AM. Conner [11] facilitated the product development decisions by presenting a reference system for describing the key attributes of a product. Thompson [12] presented the major opportunities, constraints, and economic considerations for design for AM and identified different promising directions for research and the exploitation of AM. At the same time, AM technology has also become very useful for designing new types of materials such as: meta-materials, heterogeneous materials, and cellular materials, e.g., for obtaining porous material structures. These structures have significant applications in the machine building, aerospace, and automotive industries. Türk [13] presents four design principles that improve the production of composites parts. These structures can be used in the medical field too, as long as these materials will be accepted by the body of the living organisms. In this regard, van Eijnatten [14] makes a study to assess the image quality and the accuracy of STL models acquired using different CT scanners and acquisition parameters.

The present paper reports a study on determining the compressive strength of different types of lattice cell parts, made from polymer materials. The analysed samples were made from VEROCLEAR RGD810 material, using a Connex 500 Polyjet three-dimensional printing machine. For an accurate analysis, the compressive strength of the samples was calculated in two different ways: as a ratio between the maximal supported force and the mass of the sample (N/g); as a ratio between the maximal supported force and the critical section of the sample (MPa). The results were also compared with an finite element analysis (FEA) simulation (MPa).

The results from these analyses, presented in this article, can be used to improve different structures that are used in the aerospace industry, machine building, or automotive industry.

## 2. Materials and Methods

After analysing the research conducted so far on the compressive strength of AM parts, it was found that most work had been conducted with metal parts, manufactured using selective laser melting (SLM): Maskery [15] examined the mechanical behaviour of uniform and graded density SLM Al-Si10-Mg lattices under quasistatic loading. Choy [16] explored the mechanical properties of functionally graded lattice structures fabricated by SLM, with Ti-6Al-4V as a building material. Kadkhodapour [17] developed new structure–property relations for Ti6-Al-4V scaffolds.

The mechanical strength of polymer AM parts has been investigated less often [18]. Since the manufacturing cost of parts produced by SLM is much higher than the costs of polymer three-dimensional printed parts, it was considered useful to test a wide range of cellular materials produced in this way in order to determine the best mechanical properties of the analysed structures before testing a smaller number of parts produced by SLM.

Previous studies were conducted by the authors on lattice structure parts produced using SLM in titanium alloys (Ti-6AL-4V). The influence of the lattice structures on corrosion of the microstructure and micro-hardness were analysed. Additionally, Lancea [19] presented a microstructure analysis by using scanning electron microscopy (LEO 1525 SEM) of Ti6Al4V parts exposed to a corrosive environment. Furthermore, several studies have been examined the influence of the metal powder layer thickness on the internal structure and the micro-hardness of the SLM steel parts. Buican [20] investigated the effect of building stainless steel parts, with different layer sizes and compared the micro hardness of three-dimensional metallic parts, obtained by using a laser beam that melts fine powder on a layer-by-layer basis [21].

Besides the above-mentioned articles, other studies have been conducted on lattice structure samples with different materials (steel, tool steel, aluminium and titanium alloys). In the framework of an international SFERA research project called Research about the Corrosion Resistance of Different Materials used for Building Sustainable Energy Systems, the corrosion resistance over an extended period was analysed, and the results were disseminated in an article [22].

### 2.1. The Geometry of the Samples

This study aimed to determine the highest compressive strength amongst six different lattice structures, obtained through AM. In this respect, six different kinds of lattice cells were analysed, to determine which had a better resistance to compression. In order to do this, six groups of samples were produced, each group being composed of six samples built with the same lattice structure. The compressive strength of the samples was analysed in terms of the ratio between the sample mass and the maximum loading force that the structure could support, prior to the appearance of the first signs of damage.

**Figure 1:**
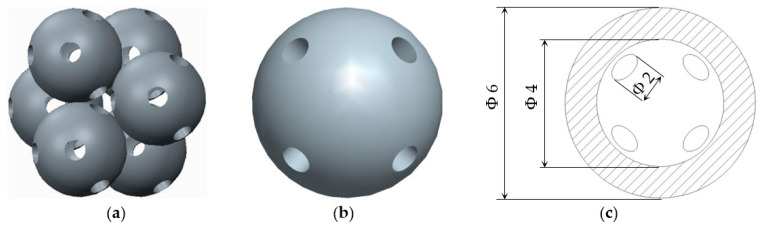
The Lattice structure (**a**) Part of the structure; (**b**) The cell; (**c**) Cell cross section.

In the following paragraphs, the six types of lattice cells that were analysed are described, each one having a structure based on spheres. In order to allow the removal of the AM support material, eight small holes were introduced into the bodies of the spherical elements (Figure 1).

**Figure 2:**
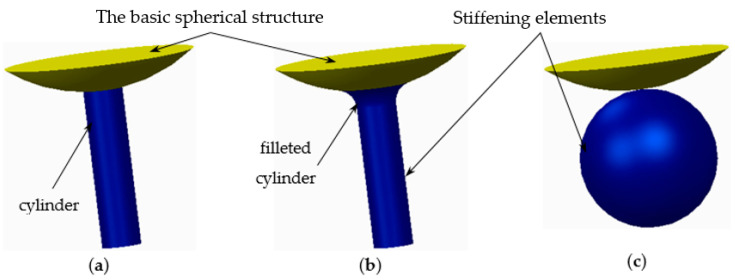
The stiffening elements (**a**) cylinder; (**b**) filleted cylinder; (**c**) sphere.

The novelty brought to these structures, aside from the eight holes, consisted of the addition of different stiffening elements placed between the basic elements, in order to increase their resistance to compression. The bonding elements have different geometries (Figure 2) and were arranged so as to create different shapes of structures to be analysed.

**Figure 3:**
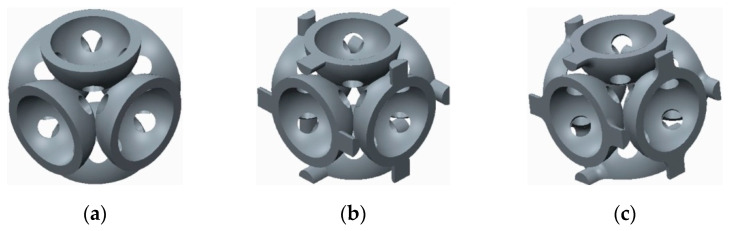
The basic structure (**a**) Without stiffening elements; (**b**) With cylindrical stiffening elements; (**c**) With filleted cylindrical stiffening elements.

**Figure 4:**
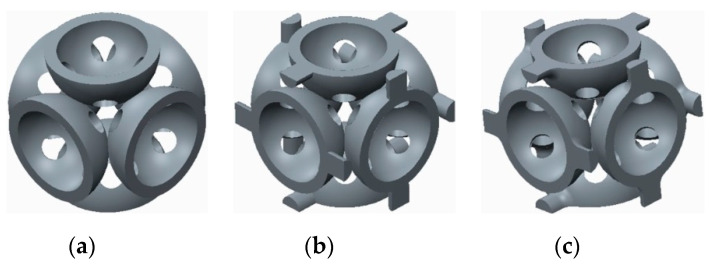
The basic structure with doubled stiffening elements (**a**) Cylinders; (**b**) Filleted cylinders; (**c**) Spherical elements.

The samples that were tested can be grouped into three categories, as follows:

- The basic structure, without stiffeners—group 1 samples (Figure 3a);
- The basic structure with stiffening elements—groups 2 and 3 samples (Figure 3b,c);
- The basic structure with doubled stiffening elements—groups 4, 5 and 6 samples (Figure 4).

### 2.2. Parts Manufacturing and the Equipment that Was Used to Perform Compression Tests

The six groups of samples were manufactured on a Connex 500 PolyJet three-dimensional printing machine using a transparent polymer composite material known as VEROCLEAR RGD810. The parts were built in high quality (HQ) printing mode, at 12 mm per hour, in 16-micron (0.0006 inch) layers thickness, having the polymerized density of 1.18 g/cm^3^ and the bulk tensile strength of 55 MPa.

**Figure 5:**
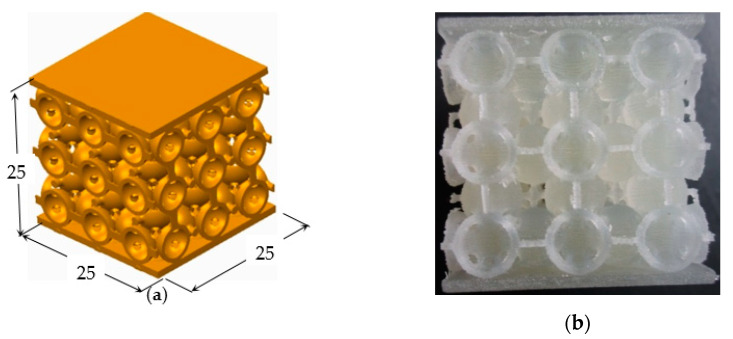
Typical sample: (**a**) CAD model; (**b**) the real part.

The dimensions of the samples (in mm) were adopted in accordance with the ASTM Standards for Polymer Composite Materials [23] (Figure 5) and the apparatus used for the compression tests was an electromechanical test machine: Instron 3366 and the related software: Bluehill 3.

## 3. Experimental Method

After manufacturing of all the samples, each one was subjected to a compression test. In order to homogenize the compression test results and to decrease the error rate, a compact “sandwich structure” was designed for all six groups of samples. In this set-up, two parallelepiped panels were attached to the top and the bottom of the lattice structure, as shown in Figure 5.

The Instron 3366 test machine and the Bluehill 3 software were used with the following parameters:

- The platform moving speed, during the tests: vpm = 6 mm/min;
- The loading force: Fl = 3000 N;
- Height of the punch, after finishing the charging amounts (above the piece): hp = 5 mm;
- The chosen load method: comprehensive Strain;
- Geometry of the samples: overall cubic shape with the dimensions: 30 mm × 30 mm × 30 mm.

**Figure 6:**
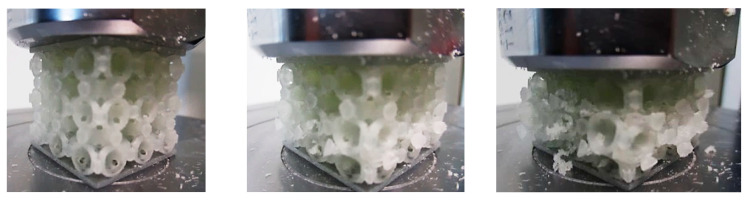
Different stages of the sample compression test.

Each sample was loaded in compression until it was completely destroyed (Figure 6).

This software package was also used also to calculate the Young’s modulus for further analysis aimed at studying the elasticity of the structures.

**Figure 7:**
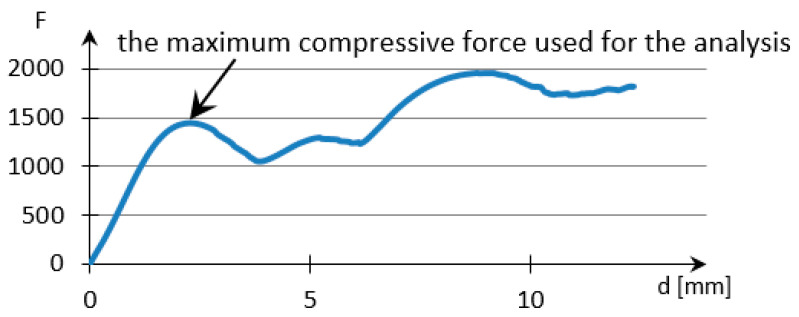
Sample compression test load versus extension graph.

The analysis of samples was completed only on the first portion of the load, until the first sign of structural damage appeared, namely until the first crack appeared on the workpiece surface. This situation is evidenced by the appearance of the first point of maximum on the loading graph. The maximum compressive force for all of the samples was taken from this position for further analysis (Figure 7).

## 4. Results

An average of all six loading graphs, for each group of samples was calculated and these are shown in Table 1.

Another important consideration for evaluation, was the average mass of the samples after all support material had been thoroughly removed.

For this, all samples in each group were weighed using the analytical balance XA 310.4Y and the average mass was calculated as an arithmetic mean.

The results of these calculations are shown in Table 2.

## 5. Discussion

In order to establish which lattice structure demonstrated the best performance under compression, the ratio between the compressive force at first sign of damage and sample mass was calculated. For this purpose, the arithmetic mean of the failure compressive forces for each group of samples was calculated. These results, together with the values for all sample masses are shown in Table 2 where:

- F_i-j_ is the maximum compressive force of the sample j that is a member of the i group;
- M_i-j_ is the mass of the sample j that is a member of the i group.

The results of these calculations were used to determine the structure that had the best compressive performance, as given by Equation (1):

(1) $${\mathrm{Rc}}_{i\;\max}=\frac{{\mathrm{Fc}}_{i\;\mathrm{med}}}{m_{i\;\mathrm{med}}}$$

where:

Rc_i max_—the compressive strength to mass ratio at initial failure
Fc_i med_—the average of compression force at initial failure
M_i med_—the average mass

By applying Equation (1) to each group of samples and combining the results presented in Table 1 and Table 2, the following results were obtained:

From these results (Table 3), the conclusion is that the lattice structure with the highest compressive strength to mass ratio is the cell that had spherical connectors where Rcmax = 192.571 (N/g).

To improve the interpretation of the results, a finite element analysis (FEA) of the samples was performed for determining the von Mises failure index [24], used to locate the areas where the greatest stresses occured. The VEROCLEAR RGD810 material was defined as a new material within the PTC Creo Parametric 2.0 CAD system based on its thermo-mechanical properties, and was assigned to all the samples.

**Figure 8:**
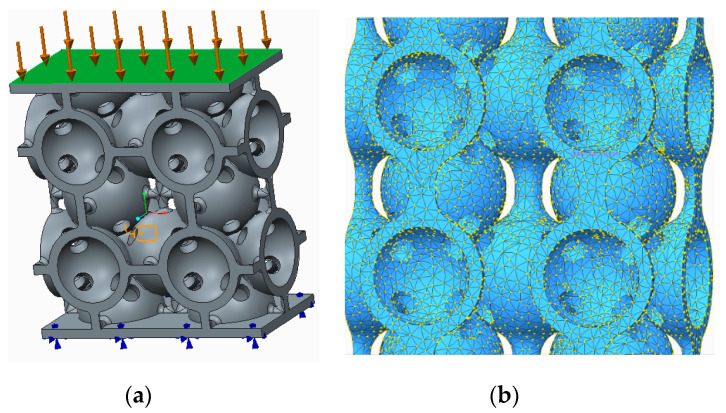
Finite element analysis (FEA) (**a**) Fixtures and external loads for FEA; (**b**) Sample mesh.

The fixtures and external loads assigned are presented in Figure 8a. All the samples were meshed into 1 mm elements, resulting in 50,413 Tetra entities for the sample presented in Figure 8b. Afterwards, the compressive strength was calculated, expressed in N/mm^2^, to be compared with the FEA results.

The simulation results were obtained using static structural analyses. The samples were fixed at the lower plane and a uniformly distributed a force of 2000 N was placed on the upper plane (Figure 8a).

**Figure 9:**
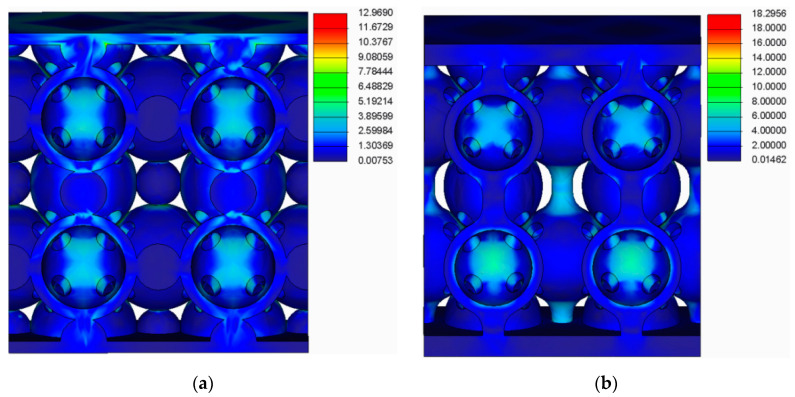
FEA results (**a**) For the samples with spherical supports (**b**) For the samples with filleted cylindrical supports.

**Figure 10:**
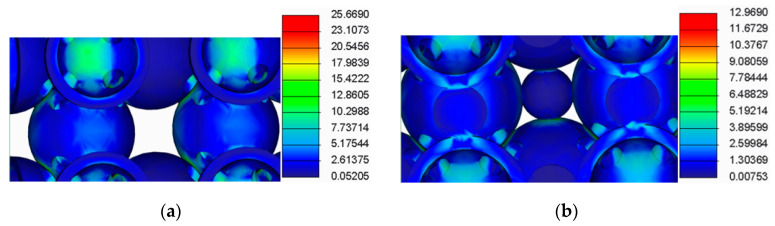
FEA detail (**a**) For samples without supports (**b**) For samples with spherical supports.

**Figure 11:**
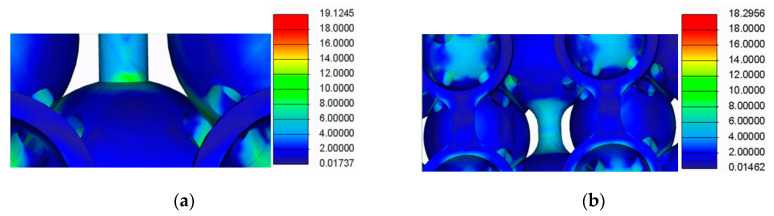
FEA detail (**a**) For the samples with cylindrical supports; (**b**) For samples with filleted cylindrical supports.

**Figure 12:**
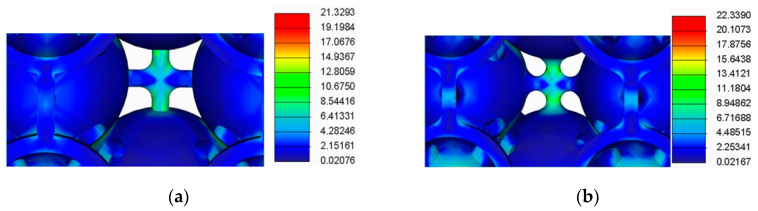
FEA detail (**a**) For the samples with doubled cylindrical supports; (**b**) For samples with doubled filleted cylindrical supports.

The results of this analysis are presented in Figure 9, Figure 10, Figure 11 and Figure 12.

By analysing the results from the FEA of the samples, several issues were observed:

- The spherical supports penetrated the spheres in the main structure of the samples;
- For the samples with double supports, the supports failed before the spheres in the main structure failed;
- The samples with a lower number of supports had a more uniform stress distribution across the supports and the main structure.

**Figure 13:**
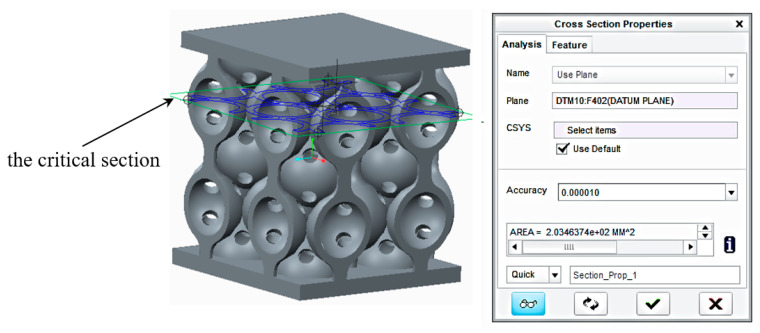
The critical section of the samples given by the FEA results.

Additionally, the FEA results showed that the most critically stressed section was the contact area between the spheres. Therefore, the samples were sectioned with a plane defined by the contact points of the spheres, and the result was a sectional area of 203.5 mm^2^ (Figure 13).

The compressive strength can be calculated using Equation (2), giving a result in N/mm^2^.

(2) $${\mathrm{Rc}}_{i\;\max}=\frac{{\mathrm{Fc}}_{i\;\mathrm{med}}}{S}$$

where:

Rc_i max_—the compressive strength to mass ratio at initial failure
Fc_i med_—the average of compression force at initial failure
S (mm^2^)—the area of the critical section.

Table 4 presents a comparison of the values for compressive strength, obtained via calculations using Equation (2), and via FEA.

Reasonably good agreement was observed between the two sets of results (within 6.1%).

## 6. Conclusions

After analysing all the results obtained within this paper, a series of conclusions can be drawn:

- The compressive strength of the spherical lattice structures increases when link elements are added;
- After analysing all the samples, it was found that the structures with spherical link elements offer the optimal structure to be used for parts subjected to compression;
- In the case of single directional loads, doubling the number of link elements had little influence on the compressive strength of the samples;
- After comparing the information obtained from the loading graphs with the FEA results, it was found that for the structures with double the number of supports, the supports crushed before the destruction of the spheres in the main structure. For the other cases (the samples with spherical supports and the samples with a smaller number of supports) the structure compactness was increased and failure of the supports occurred simultaneously with the failure of the spheres in the main structure;
- Due to the much lower manufacturing cost of the polymer parts, compared to the price of the metal alloy parts, the results of these tests can be an important starting point for substantially reducing the testing costs of the metal parts, produced by SLM technology.

Although a rather narrow range of lattice structure designs was used for this work, the authors believe that the methods used and the lessons learnt will be applicable to a wider set of lattice structure designs.

## Figures and Tables

**Table 1:** The compressive resistance and the average mass of the samples.

Structures with Stiffening Elements		Structures with Doubled Stiffening Elements	
Lattice cell geometry	The loading force (N) × displacement (mm) graph	Lattice cell geometry	The loading force (N) × displacement (mm) graph
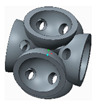 Without stiffening supports	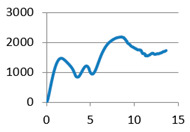	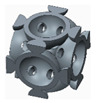 With spherical supports	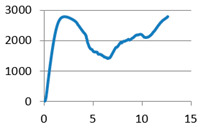
	Part mass average: 11.82 g		Part mass average: 13.601 g
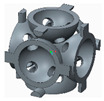 With cylindrical supports	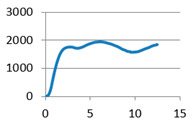	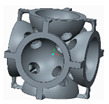 With cylindrical supports	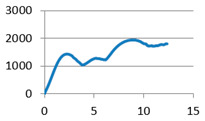
	Part mass average: 12.225 g		Part mass average: 12.018 g
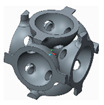 With filleted cylindrical supports	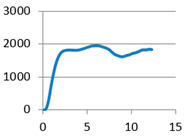	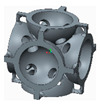 With doubled filleted cylindrical supports	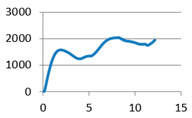
	Part mass average: 12.306 g		Part mass average: 12.044 g

**Table 2:** The failure compressive force for each sample together with the sample masses.

Lattice Cell Geometry	Maximum Compressive Force (N)	Mass (g)	Lattice Cell Geometry	Maximum Compressive Force (N)	Mass (g)
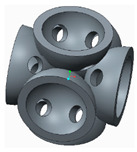 Without stiffening supports	F_1-1_: 1415.089 F_1-2_: 1404.516 F_1-3_: 1456.453 F_1-4_: 1147.601 F_1-5_: 1311.217 F_1-6_: 1338.975	m_1-1_: 12.172 m_1-2_: 11.703 m_1-3_: 12.117 m_1-4_: 11.951 m_1-5_: 11.610 m_1-6_: 11.365	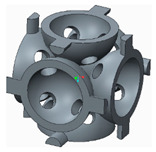 With cylindrical supports	F_2-1_: 1696.994 F_2-2_: 1783.833 F_2-3_: 1896.945 F_2-4_: 1765.031 F_2-5_: 1698.578 F_2-6_: 1782.309	m_2-1_: 12.725 m_2-2_: 12.058 m_2-3_: 11.512 m_2-4_: 11.886 m_2-5_: 12.618 m_2-6_: 12.548
**Average value**	**1345.642**	**11.82**	**Average value**	**1770.615**	**12.225**
**Standard deviation**	**110.47**	**0.314**	**Standard deviation**	**73.364**	**0.482**
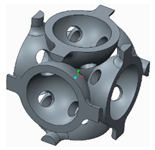 With filleted cylindrical supports	F_3-1_: 2120.697 F_3-2_: 1779.39 F_3-3_: 1808.261 F_3-4_: 1863.207 F_3-5_: 1710.604 F_3-6_: 1886.721	m_3-1_: 12.586 m_3-2_: 12.042 m_3-3_: 12.305 m_3-4_: 11.852 m_3-5_: 12.801 m_3-6_: 12.250	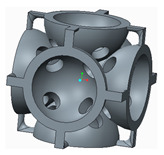 With doubled cylindrical supports	F_4-1_: 1523.174 F_4-2_: 1703.174 F_4-3_: 1655.9434 F_4-4_: 1591.677 F_4-5_: 1442.222 F_4-6_: 1495.113	m_4-1_: 11.479 m_4-2_: 12.254 m_4-3_: 12.518 m_4-4_: 12.650 m_4-5_: 11.687 m_4-6_: 11.520
**Average value**	**1861.48**	**12.306**	**Average value**	**1568.55**	**12.018**
**Standard deviation**	**141.54**	**0.347**	**Standard deviation**	**99.706**	**0.52**
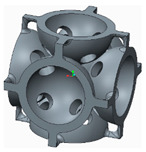 With doubled filleted cylindrical supports	F_5-1_: 1568.214 F_5-2_: 1552.337 F_5-3_: 1631.017 F_5-4_: 1607.449 F_5-5_: 1651.441 F_5-6_: 1593.386	m_5-1_: 12.215 m_5-2_: 12.399 m_5-3_: 12.541 m_5-4_: 11.364 m_5-5_: 11.511 m_5-6_: 12.231	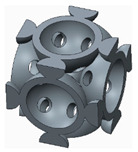 With spherical supports	F_6-1_: 2619.124 F_6-2_: 2644.336 F_6-3_: 2603.658 F_6-4_: 2795.897 F_6-5_: 2567.721 F_6-6_: 2484.217	m_6-1_: 14.379 m_6-2_: 12.789 m_6-3_: 14.192 m_6-4_: 13.755 m_6-5_: 12.594 m_6-6_: 13.899
**Average value**	**1600.641**	**12.044**	**Average value**	**2619.159**	**13.601**
**Standard deviation**	**37.382**	**0.487**	**Standard deviation**	**102.9223**	**0.740**

**Table 3:** The compressive strength obtained with Equation (1).

Samples Type	Rc_imax_ (N/g)
Samples without supports	Rc_1max_ = 1345.642/11.82 = 113.845 N/g
Samples with spherical supports	Rc_2max_ = 2619.159/13.601 = 192.571 N/g
Samples with cylindrical supports	Rc_3max_ = 1770.615/12.225 = 144.836 N/g
Samples filleted cylindrical supports	Rc_4 max_ = 1861.48/12.306 = 151.266 N/g
Samples doubled cylindrical supports	Rc_5 max_ = 1568.55/12.018 = 130.517 N/g
Samples doubled filleted cylindrical supports	Rc_6 max_ = 1600.641/12.044 = 132.899 N/g

**Table 4:** The compressive strength values obtained via calculation and FEA.

Samples Type	Calculated Rc_imax_ (MPa)	Rc_imax_ by FEA (MPa)
Samples without supports	Rc_1max_ = 25.05	Rc_1max_ = 25.67
Samples with spherical supports	Rc_2max_ = 12.87	Rc_2max_ = 12.97
Samples with cylindrical supports	Rc_3max_ = 19.04	Rc_3max_ = 19.12
Samples filleted cylindrical supports	Rc_4 max_ = 18.11	Rc_4 max_ = 18.30
Samples doubled cylindrical supports	Rc_5 max_ = 21.49	Rc_5 max_ = 21.33
Samples doubled filleted cylindrical supports	Rc_6 max_ = 21.06	Rc_6 max_ = 22.34
